# Impact of Acceptance and Commitment Therapy (ACT) on Psychological Flexibility in Medical Students

**DOI:** 10.1192/j.eurpsy.2025.2140

**Published:** 2025-08-26

**Authors:** E. Cetintas, M. E. Karadere, F. Cetinkaya

**Affiliations:** 1 Istanbul Medeniyet University, Department of Psychiatry, Istanbul, Türkiye

## Abstract

**Introduction:**

Medical school is known for high levels of anxiety, and mental health challenges. Psychological flexibility - the ability to accept difficult thoughts and emotions while acting in alignment with one’s values- can protect against these challenges. ACT has been shown to enhance psychological flexibility and reduce mental health symptoms in various populations. However, its impact on medical students has not been extensively explored.

**Objectives:**

This study aimed to assess whether a brief ACT intervention could improve psychological flexibility and related psychological outcomes in medical students.

**Methods:**

Forty-two medical students from Istanbul Medeniyet University participated in a four-day ACT program. Inclusion criteria included active enrollment in a medical program and willingness to participate. Exclusion criteria were severe psychiatric disorders or ongoing psychiatric treatment.The intervention involved four days of ACT-based activities, including mindfulness, cognitive defusion, and value-based exercises. Each session lasted 90 minutes and was conducted in a group format. To assess the impact of the intervention, we measured psychological flexibility and related factors at baseline and after the program using the following tools: the Multidimensional Experiential Avoidance Questionnaire (MEAQ-30), AAQ-US, SF-25, Valuing Questionnaire (VQ) and the DASS-21. Paired t-tests were used to compare pre- and post-intervention scores, with statistical significance set at p < 0.05.

**Results:**

The ACT intervention led to significant improvements in psychological flexibility and related factors. The ACT intervention led to significant improvements in psychological flexibility and related factors. Specifically, Distress Aversion (MEAQ-30, subscale B) decreased significantly (p = 0.010), while Distress Endurance (MEAQ-30, subscale F) increased significantly (p = 0.008). Additionally, Anxiety (DASS-A) decreased significantly (p = 0.004), and Stress (DASS-S) also decreased significantly (p = 0.004). No significant changes were observed in other subscale scores, including those for SF-36, depression (DASS-D), and scores for AAQ-US and VQ. These results suggest that ACT may be particularly effective in reducing anxiety and stress while enhancing cognitive flexibility and acceptance.

**Conclusions:**

This study suggests that even a brief, four-day ACT intervention can significantly improve psychological flexibility and reduce anxiety and stress in medical students. These findings highlight ACT’s potential as an effective tool for supporting mental health and resilience in medical students. Further research with larger sample sizes and longer follow-up periods is needed to explore the long-term impact of ACT interventions in academic settings.

**Disclosure of Interest:**

None Declared

